# Effects of Interscalene Nerve Block for Postoperative Pain Management in Patients after Shoulder Surgery

**DOI:** 10.1155/2015/902745

**Published:** 2015-11-24

**Authors:** Hsiu-Pin Chen, Shih-Jyun Shen, Hsin-I Tsai, Sheng-Chin Kao, Huang-Ping Yu

**Affiliations:** ^1^Department of Anesthesiology, Chang Gung Memorial Hospital, Taoyuan 333, Taiwan; ^2^College of Medicine, Chang Gung University, Taoyuan 333, Taiwan

## Abstract

*Objectives*. Shoulder surgery can produce severe postoperative pain and movement limitations. Evidence has shown that regional nerve block is an effective management for postoperative shoulder pain. The purpose of this study was to investigate the postoperative analgesic effect of intravenous patient-controlled analgesia (PCA) combined with interscalene nerve block in comparison to PCA alone after shoulder surgery.* Methods*. In this study, 103 patients receiving PCA combined with interscalene nerve block (PCAIB) and 48 patients receiving PCA alone after shoulder surgery were included. Patients' characteristics, preoperative shoulder score and range of motion, surgical and anesthetic condition in addition to visual analog scale (VAS) pain score, postoperative PCA consumption, and adverse outcomes were evaluated.* Results*. The results showed that PCA combined with interscalene nerve block (PCAIB) group required less volume of analgesics than PCA alone group in 24 hours (57.76 ± 23.29 mL versus 87.29 ± 33.73 mL, *p* < 0.001) and 48 hours (114.86 ± 40.97 mL versus 183.63 ± 44.83 mL, *p* < 0.001) postoperatively. The incidence of dizziness in PCAIB group was significantly lower than PCA group (resp., 1.9% and 14.6%, *p* = 0.005). VAS, nausea, and vomiting were less in group PCAIB, but in the absence of significant statistical correlation.* Conclusion*. Interscalene nerve block is effective postoperatively in reducing the demand for PCA analgesics and decreasing opioids-induced adverse events following shoulder surgery.

## 1. Introduction

As the shoulder is the complex, mobile, and inherently unstable joint in the body, it can get injured easily. Shoulder pain is in charge of approximately 16 to 20% of all musculoskeletal complaints [1], with a yearly incidence of 1% new episodes in general population [2]. If pain is not adequately treated, it can result in the sensitization of the peripheral and central nervous system and lead to the development of chronic pain [3–5].

Severe postoperative pain is often accompanied with major shoulder surgery [6]. Therefore, without large-dose opioids, the pain could be difficult to control [7, 8]. Opioids can cause many adverse outcomes, such as nausea, vomiting, dizziness, physical dependence, and unexpected sedation [8–10].

An interscalene nerve block provides not only excellent muscle relaxation, but also a well-recognized and efficient technique for pain management [11]. This paper hypothesized that the postoperative pain control by supplementing intravenous patient-controlled analgesia (PCA) with interscalene nerve block may provide better pain control and decrease total amount of analgesics consumption compared with continuous PCA infusion alone.

## 2. Material and Methods

### 2.1. Data Sources and Study Population

We acquired data from Chang Gung Memorial Hospital's Pain Service database that included patient demographics, diagnosis of diseases, surgical procedures, medication, and medical expenditures. The study was approved by the ethical review committee of Chang Gung Memorial Hospital in accordance with the 2008 Declaration of Helsinki.

From January 2007 to December 2013, 151 patients received PCA after elective shoulder surgeries. The demographics of patients, such as age and weight, and preoperative medical history including hypertension, arrhythmia, asthma, hepatitis, gastric ulcer, renal syndrome, diabetes mellitus, cerebral embolism, myocardial infarction, and cigarette smoking status were collected from the database. Preoperative shoulder condition including University of California at Los Angeles (UCLA) preoperative shoulder score, range of motion in forward elevation, and external rotation as well as intraoperative surgical and anesthetic time was also recorded.

### 2.2. Procedure of Anesthesia and Postoperative Analgesia Procedure

All of the 151 patients received general anesthesia. After reaching postanesthetic care unit (PACU), 1 mcg/kg fentanyl was first injected intravenously, and a loading dose was then given. The content of the PCA bag was constituted of 1000 mcg fentanyl and 300 mg ketorolac with normal saline to a volume of 330 mL in total. The PCA program was set up as a loading dose of 2 mL, an infusion rate of 2 mL/hr, breakthrough bolus of 3 mL, and lockout time at 5 minutes. All patients received 8 mg ondansetron as preventive antiemetics.

The interscalene block was performed by positioning the patients lying supine, head slightly elevated and turned away from the side to be blocked. The patients were given mild sedation with 0.05 mg/kg midazolam in an attempt to maintain verbal contact. Appropriate aseptic precautions were taken. A linear ultrasound probe (frequency 10–15 MHz) was used with the depth setting of 2–4 cm. The probe was initially placed near the midline of clavicle at the level of cricoid cartilage and scanned laterally to identify the carotid artery and internal jugular vein underneath the sternocleidomastoid muscle. By moving the probe laterally, the anterior scalene muscle was seen below the lateral edge of the sternocleidomastoid. A groove containing the hypoechoic nerve structures could usually be identified. 20 mL of 0.25% levobupivacaine was used for nerve paraesthesia blockade.

The intensity of pain was assessed using a visual analogue scale (VAS) based on a total score between 0 and 10 (0 = no pain and 10 = worst pain). Patients were discharged from PACU when fulfilling discharge criteria: Steward Score was greater than 4 and VAS for pain was less than 4.

### 2.3. Outcome Measures

The primary outcome was the total amount of intravenous PCA analgesics used. Secondary outcomes included postoperative VAS and adverse effects.

### 2.4. Statistical Analysis

Data were collected and expressed as number, percentage, and mean ± standard deviation. The statistical result of pain scores was expressed as median with interquartile range. Normally distributed data were compared between groups using the unpaired Student's *t*-test, and continuous variables with a non-Gaussian distribution were presented as median with ranges and compared between groups using Mann-Whitney *U* test. Group differences with nominal variables were analyzed using Chi-square or Fisher's exact tests for proportions. A *p* value < 0.05 was considered to be statistically significant. All statistical data were analyzed using the SPSS statistical software version 19.0 for Windows (SPSS Inc., Chicago, IL, USA).

## 3. Results

### 3.1. Study Cohort

The total cohort consisted of 151 surgical patients receiving PCA after undergoing shoulder surgery. The eligible study subjects were 103 surgical patients with single bolus interscalene block with PCA (group PCAIB) and 48 surgical patients receiving intravenous PCA only (group PCA). No significant difference in patients' characteristics and preoperative comorbidity between the groups was observed. Furthermore, preoperative shoulder condition including UCLA preoperative shoulder score, range of motion, surgical time, and anesthetic time perioperatively also showed no statistically significant differences (Tables 1 and 2).

### 3.2. Outcomes and Estimation

Surgical patients in group PCAIB required less volume of analgesics than group PCA not only in the first 24 hours postoperatively (57.8 ± 23.3 mL versus 87.3 ± 33.7 mL, *p* < 0.001) but also in the 48 hours postoperatively (114.9 ± 41.0 mL versus 183.6 ± 44.8 mL, *p* < 0.001) (Table 3). The VAS recording over the follow-up period was illustrated in Table 4. Average or worst VAS did not differ significantly between the two groups.

There was a significant difference in the incidence of dizziness between the two groups. The incidence of dizziness in group PCAIB was lower than group PCA (resp., 1.9% and 14.6%, *p* = 0.005). The incidence of nausea in group PCAIB and group PCA showed 2.9% and 10.4%, respectively, with *p* = 0.110, and the incidence of vomiting revealed 1.9% and 6.3%, respectively, with *p* = 0.327.

## 4. Discussion

A number of studies have assessed postoperative pain severity by comparing regional block with general anesthesia [7, 8, 10–16]. Previous studies were conducted using different types of regional blocks [7, 13] or at different time points in relation to the surgeries [10–12, 14, 16]. In the study, we evaluated patients receiving interscalene nerve block postoperatively at PACU in combination with intravenous PCA for postoperative pain management. Peripheral nerve blocks can be achieved with the aid of ultrasound or neurostimulation. We chose ultrasound-guided technique because postoperative neurological symptoms were not uncommon after interscalene block. Such complications can be avoided by performing nerve blocks under ultrasound guidance [17–20].

With interscalene nerve block, lower incidence of adverse effects such as nausea and vomiting, pruritus, sleep disturbance, and constipation for shoulder surgery was observed [21]. The incidence of nausea and vomiting was lower in group PCAIB in our study, although there was no significant difference between the two groups. One reason could be that a preemptive antiemetic, ondansetron, was used [22]. In addition, the regimen of PCA was constituted of fentanyl and ketorolac, which might cause less nausea and vomiting [23, 24].

No severe complications following interscalene nerve block were found in our study. However, it is important to note that some severe complications after interscalene nerve block have been reported. Ward [25] reported an incidence of 3% of symptomatic pneumothorax after interscalene nerve block. Another rare but severe complication after interscalene nerve block, persistent phrenic palsy, can potentially be life-threatening, especially in patients with previous lung function impairment [26].

There are some limitations in our study. At first, this is a retrospective, nonrandomized study, which introduces inherent biases shared by all retrospective studies. The number of patients between two groups could not be assigned equally to two groups when extracting data form Chang Gung Memorial Hospital Pain Service database. Secondly, selection bias was introduced as patients may prefer one postoperative pain management over the other. Despite these limitations, our study might present insight into interscalene nerve block in postoperative pain management for shoulder surgery.

## 5. Conclusion

In conclusion, interscalene nerve block could significantly reduce postoperative PCA narcotic requirement and decrease opioids-induced adverse events (Table 5) following shoulder surgery. Interscalene nerve block might provide ideal pain management after shoulder surgery. However, some rare but major complications of interscalene nerve block still need to be kept in mind.

## Figures and Tables

**Table 1:** General characteristics of patients.

Parameters	PCAIB group	PCA group	*p*
Gender			0.595
Female	32 (31.%)	17 (35%)	
Male	71 (69%)	31 (65%)	
Age (year)	59.4 ± 12.2	57.8 ± 12.1	0.442
Weight (kg)	63.2 ± 11.7	63.6 ± 12.6	0.826
Surgical procedure			0.727
Open	42 (41%)	21 (44%)	
Arthroscopy	61 (59%)	27 (56%)	
Surgical time (min)	84 ± 25	77 ± 27	0.147
Anesthesia time (min)	143 ± 44	132 ± 31	0.115
American Society of Anesthesiologists (ASA) physical status			0.823
I	18 (18%)	7 (15%)	
II	73 (71%)	34 (71%)	
III	12 (12%)	7 (15%)	
Preoperative evaluation			
UCLA preoperative score	8.2 ± 3.1	8.1 ± 2.5	0.873
Preoperative forward elevation (deg.)	87.5 ± 19.1	90.0 ± 20.6	0.665
Preoperative external rotation (deg.)	42.9 ± 4.9	42.7 ± 5.1	0.795

Continuous variables were described as the mean ± standard deviation, and the categorical variable was described as number of events (*n*/%); the remaining parameters were compared using an independent *t*-test, and statistical significance was considered when *p* < 0.05.

UCLA preoperative score: University of California at Los Angeles Shoulder Score.

PCAIB: patients with interscalene block in combination with intravenous patient-controlled analgesia.

PCA: patients receiving intravenous patient-controlled analgesia alone without interscalene block.

**Table 2:** Preoperative comorbidities.

Preoperative comorbidity	PCAIB group	PCA group	*p*
Cardiovascular system			
Hypertension	42 (41%)	21 (44%)	0.726
Arrhythmia	9 (9%)	9 (19%)	0.105
Respiratory system			
Asthma	6 (6%)	4 (8%)	0.727
Gastrointestinal system			
Hepatitis	13 (13%)	7 (15%)	0.741
Gastric ulcer	13 (13%)	6 (13%)	0.983
Urologic system			
Renal syndrome	4 (4%)	1 (2%)	1.000
Endocrine system			
DM	19 (18%)	8 (17%)	0.790
Thromboembolic events			
Cerebral embolism	2 (2%)	1 (2%)	1.000
Myocardial infarction	3 (3%)	2 (4%)	0.653
Smoking	64 (62%)	30 (63%)	0.966

Categorical variables as number of events (*n*); Chi-square test was used, events less than 5 were compared with Fisher's exact test, and statistical significance was considered when *p* < 0.05.

PCAIB: patients with interscalene block in combination with intravenous patient-controlled analgesia.

PCA: patients receiving intravenous patient-controlled analgesia alone without interscalene block.

**Table 3:** Total amount of patient-controlled analgesia and rescue medications.

	PCAIB group	PCA group	*p*
Amount of medications (mL)			
24 hours postoperatively	57.76 ± 23.29	87.29 ± 33.73	<0.001^*∗*^
48 hours postoperatively	114.86 ± 40.97	183.63 ± 44.83	<0.001^*∗*^

Continuous variables were described as mean ± standard deviation and independent *t*-test was used; ^*∗*^statistical significance was considered when *p* < 0.05.

PCAIB: patients with interscalene block in combination with intravenous patient-controlled analgesia.

PCA: patients receiving intravenous patient-controlled analgesia alone without interscalene block.

**Table 4:** Visual analog pain score.

	PCAIB group	PCA group	*p*
Median of average VAS at 24 h	2 (0–3)	2 (0–3)	0.985
Median of worst VAS at 24 h	3 (2–5)	4 (2–6)	0.229
Median of average VAS at 48 h	3 (2–5)	4 (2–6)	0.548
Worst VAS at 48 h	4 (3–6)	5 (2–8)	0.185

Data are presented as median with interquartile range.

Visual analog scale (VAS) score (0 = no pain; 10 = worst pain).

PCAIB: patients with interscalene block in combination with intravenous patient-controlled analgesia.

PCA: patients receiving intravenous patient-controlled analgesia alone without interscalene block.

**Table 5:** Adverse events.

Adverse events	PCAIB group	PCA group	*p*
Dizziness	2 (2%)	7 (15%)	0.005^*∗*^
Nausea	3 (3%)	5 (10%)	0.110
Vomiting	2 (2%)	3 (6%)	0.327
Respiratory depression	0	0	—
Skin itching	0	0	—
Urine retention	0	0	—
Muscle weakness	0	—	—
Numbness	3 (3%)	—	—
Hemidiaphragmatic paresis	0	—	
Local hematoma or infection	0	—	—

Categorical variables as number of events (*n*); Chi-square test was used, events less than 5 were compared with Fisher's exact test, and ^*∗*^statistical significance was considered when *p* < 0.05.

PCAIB: patients with interscalene block in combination with intravenous patient-controlled analgesia.

PCA: patients receiving intravenous patient-controlled analgesia alone without interscalene block.
